# Tuberculosis and HIV co-infection: its impact on quality of life

**DOI:** 10.1186/1477-7525-7-105

**Published:** 2009-12-29

**Authors:** Amare Deribew, Markos Tesfaye, Yohannes Hailmichael, Nebiyu Negussu, Shallo Daba, Ajeme Wogi, Tefera Belachew, Ludwig Apers, Robert Colebunders

**Affiliations:** 1 Department of Epidemiology, Jimma University, Jimma, Ethiopia; 2 Department of Psychiatry, Jimma University, Jimma, Ethiopia; 3 Department of Health Service Management, Jimma University, Jimma, Ethiopia; 4 Malaria Control Program, Somali regional Health Bureau, Jijiga, Ethiopia; 5 HIV Prevention and Control office, Oromiya Regional Health Bureau, Addis Ababa, Ethiopia; 6 Department of Population and Family Health, Jimma University, Jimma, Ethiopia; 7 Department of Clinical Sciences, Institute of Tropical Medicine, Nationalestraat 155, 2000 Antwerp, Belgium; 8 Department of Epidemiology and Social Medicine, University of Antwerp, Campus Drie Eiken, Universiteitsplein 1 2610 Antwerpen, Belgium

## Abstract

**Background-:**

Very little is known about the quality of life of tuberculosis (TB) and HIV co-infected patients. In this study in Ethiopia, we compared the quality of life HIV positive patients with and without TB.

**Methods-:**

A cross sectional study was conducted from February to April, 2009 in selected hospitals in Oromiya Regional state, Ethiopia. The study population consisted of 467 HIV patients and 124 TB/HIV co-infected patients. Data on quality of life was collected by trained nurses through face to face interviews using the short Amharic version of the World Health Organization Quality of Life Instrument for HIV clients (WHOQOL HIV). Depression was assessed using a validated version of the Kessler scale. Data was collected by trained nurses and analyzed using SPSS 15.0 statistical software.

**Results:**

TB/HIV co-infected patients had a lower quality of life in all domains as compared to HIV infected patients without active TB. Depression, having a source of income and family support were strongly associated with most of the Quality of life domains. In co-infected patients, individuals who had depression were 8.8 times more likely to have poor physical health as compared to individuals who had no depression, OR = 8.8(95%CI: 3.2, 23). Self-stigma was associated with a poor quality of life in the psychological domain.

**Conclusion-:**

The TB control program should design strategies to improve the quality of life of TB/HIV co-infected patients. Depression and self-stigma should be targeted for intervention to improve the quality of life of patients.

## Background

Ethiopia is among the countries most heavily affected by the Human immunodeficiency Virus (HIV) and tuberculosis (TB). There are an estimated 1.3 million people living with the virus and roughly 68,136 of them were children under 15 years [1]. The World Health Organization (WHO) has classified Ethiopia 7^th ^among the 22 high burden countries with TB and HIV infection in the world [2]. The annual TB incidence of Ethiopia is estimated to be 341/100,000. TB mortality rate is 73/100,000 and the prevalence of all forms TB is estimated to be 546/100,000 [3]. About 40-70% of HIV patients in Ethiopia are co-infected with TB [4,5].

TB and HIV co-infection are associated with special diagnostic and therapeutic challenges and constitute an immense burden on healthcare systems of heavily infected countries like Ethiopia [1,2]. In Ethiopia, free Antiretroviral therapy (ART) has been given to patients since 2003[6]. Directly observed therapy short course therapy (DOTS) for TB patients has been operational since 1993 in Ethiopia [7]. To improve quality of life (QOL), it is crucial to identify the determinants QOL. Worldwide, many QOL studies have been performed among patients with HIV infection [8-13] and among patients with TB [14-18]. However, there is dearth of literature on the QOL of TB/HIV co-infected patients. In this study in Ethiopia, we compare the QOL of HIV infected patients with and without active TB.

## Methods

### Study Settings and Population

From February to April, 2009, we conducted a cross sectional study in three hospitals in Oromiya regional state of Ethiopia. Based on the availability of patients, we selected Adama, Nekemet and Jimma specialized hospitals in the east, west and southwest part of Ethiopia respectively. The study population consisted of HIV patients with and without active TB who had regular follow up in the TB/HIV clinics of these hospitals for the last one year. Sample size for the two groups was determined using WINPEPI (Window program for Epidemiologist) [19]. In a recent study, the mean score of general QOL among HIV infected patients who were taking highly active antiretroviral therapy in Jimma hospital was 87 [20]. Due to absence of data, we assumed HIV/TB co-infected patients would have a 5% lower mean score of general health as compared to HIV patients. With a power of 80%, 95% CI, a 1:3 ratio of HIV/TB co-infected patients versus HIV patients, and a 10% for non-response rate, the sample size was 620 (155 co-infected patients and 465 HIV patients). During the study period, all new TB patients were indentified among the HIV clients who regularly attended the HIV clinics. Only patients who were in the intensive phase of anti-TB treatment during the study period were included. For each TB/HIV co-infected patients, 3 HIV patients without active TB were selected in the TB/HIV clinics using a simple random sampling technique. The exclusion criteria for both groups were age less than 15 years, the presence of an opportunistic infection or a known chronic illness like diabetes mellitus and hypertension. The sample size in each study group and study setting is described below (Table 1).

**Table 1:** Selection of the study participants in three hospitals in the Oromiya regional state of Ethiopia April, 2009

Hospital	Number on Antiretroviral therapy	Randomly selected HIV patients	Number of TB/HIV co-infected
Adama	4616	304	101
Jimma	1252	82	27
Nekemet	1185	78	26

Total	7053	465	155

### Measurements

The KHB (Shanghi Kehua Bio-engineering, Ltd, 2008, China) HIV test was used to diagnose HIV. For positive results, confirmation was done using the STAT-PAK test (Chembio diagnostic System Inc, 2008, USA). Smear microscopy was the major diagnostic tool for pulmonary TB. Patients who had two smear positive results were labeled as smear positive. Patients who had three negative smears and suggestive X-ray findings or failure to respond to an antibiotic trials were labeled as smear negative pulmonary TB. TB lymphadenitis was diagnosed based on clinical parameters and cytological examination obtained by fine needle aspiration. Data on QOL was collected by trained nurses through face to face interview using the short Amharic version of the World Health Organization Quality of Life Instrument for HIV clients (WHOQOL HIV) [8]. This instrument was used previously in Ethiopia [21] and contains 31 items. For each item there is a five-point Likert scale where 1 indicates low or negative perceptions and 5 high or positive perceptions. These items contain six domains: Physical health (4 items); psychological well being (5 items); social relationship (4 items); environmental health (8 items); level of independence (4 items) and spiritual health (4 items). There were two general questions about general QOL and perceived general health. The physical domain contained information regarding presence of pain, energy and sleep. The psychological domain consisted of negative and positive feelings, self esteem and thinking. The social domain covered social support, personal relationships and sexual activity. Mobility, work capacity, and activities were included in the level of dependence. Financial issues; home and physical environment; availability of transport; physical safety and security, and participation in leisure activities were included under the environmental domain. The spirituality domain did contain questions about death and dying; forgiveness and blame and concern about the future. We also incorporated variables related to socio-demographic factors, having source of income and family support into the QOL instrument.

Depression was measured using the Kessler 10 scales [22]. This instrument has 10 questions each containing 5-point Likert scales (1 = never, 2 = a small part of the time, 3 = some of the time, 4 = most of the times, 5 = all of the time). The Kessler-10 scale was validated in Ethiopia and used extensively [23,24]. Perceived stigma was measured by 23 questions adopted from Berger et al [25]. A detailed description of the instrument is available elsewhere [unpublished manuscript by the same authors]. The stigma items consisted of four-point Likert scale (strongly disagree, disagree, agree, strongly agree) questions. Questions were asked about perceived isolation, shame, guilt and disclosure of the HIV status. Clinical information such as CD4 lymphocyte count and WHO staging were extracted from medical charts in the ART clinics.

### Data Analysis

Data were analyzed using the SPSS version 15.0 software. Domain scores in the WHOQOL-HIV were scaled in positive direction with higher score denoting good quality of life. Negative questions like pain and discomfort were recorded so that higher scores reflected better QOL. Mean scores of items within each domain were used to calculate the domain score. Mean scores were then multiplied by 4 in order to make domain scores comparable with the scores used in the World Health Organization Quality of Life instrument (WHOQOL-100). We used t-test and F-test to compare means between groups. By taking the mean or the median of each domain as a cutoff point, QOL was dichotomized as poor or good. Mean was used as a cutoff point for psychological, level of independence, social, and environmental domains (because of a normal distribution of the scores). Median was used as cutoff point for physical and spiritual domains of QOL (because of a skewed distribution of the scores). Individuals who scored below the mean/median were classified as having poor QOL. To assess predictors of QOL (poor vs. good), we first performed a bivariate analysis. Educational status, occupation, WHO staging, having a source of income, depression and perceived stigma did show statistically significant association (P < 0.05) with QOL in the bivariate analysis. These variables were entered into a stepwise logistic regression model.

### Ethical consideration

Ethical clearance was obtained from the Jimma University ethical review board. Written informed consent was obtained from the study participants. To ensure confidentiality, we used codes to analyze the data.

## Result

### Characteristics of the study participants

Of the 620 patients asked to participate in the study, 591 (95%) accepted of whom 124 (21%) were TB/HIV co-infected. Twenty nine participates refused to participate in the study. Of the co-infected patients, 61(49.2%) were smear negative, 42(33.8%) smear positive and 21(17%) extrapulmonary TB patients.

Illiterates and males were more likely to have active TB than their counter parts (P < 0.05). Co-infected patients were more likely to have a lower CD4 lymphocyte count than HIV patients (P = 0.001). All HIV patients and 75% of the co-infected patients were taking ART during the survey (Table 2).

**Table 2:** Socio-demographic and clinical characteristics of the study population in three hospitals of the Oromiya region, Ethiopia.

Variables	TB/HIV coinfected patients (N = 124) Number (%)	HIV patients (N = 467) Number (%)	P-Value
**Age in Years**			
15-24	12(9.7)	42(9.0)	0.61
25-34	53(42.7)	223(47.8)	
> = 35	59(47.6)	202(43.2)	
**Sex**			
Male	62(50)	185(39.6)	0.03
Female	62(50)	282(60.4)	
**Educational status**			
Illiterate	30(24.2)	74(15.8)	0.03
literate	94(75.8)	393(84.2)	
**Occupation**			
Government employee	17(13.7)	52(10.9)	0.003
Private employee	15(12.1)	84(18.0)	
Merchant	1(8.1)	69(14.8)	
Farmer	13(10.5)	32(6.9)	
Housewives	15(12.1)	79(17.0)	
Daily laborer	22(17.7)	93(20.0)	
No Job	32(25.8)	58(12.4)	
**WHO staging**			0.001
Stage II	13(10.6)	136(29.5)	
Stage III	96(78%)	259(56.2)	
Stage IV	14(11.4)	66(13.4)	
**CD4 lymphocyte count**			
<200	46(57.5)	112(27.3)	0.001
> = 200	34(42.5)	299(72.7)	
**On antiretroviral therapy**			0.001
Yes	93(75.6)	464(100)	
NO	30(24.4)	0	

### The Kessler Scale and the stigma instrument

The correlation between items in the Kessler scale ranged from 0.5 to 0.79 with no multicollinearity and redundancy. The internal consistency of the Kessler scale was high (Cronbach's α = 0.93). Confirmatory factor analysis showed that correlation between stigma items ranged 0.49 to 0.75. There was strong correlation between stigma scales and depression (P < 0.05). Cronbach's alpha for the stigma scales ranged from 0.71 to 0.88.

### Internal consistency of the WHOQOL-HIV

To measure internal consistency, the Cronbach's alpha was calculated for each domain of the instrument. Most domains of the Amharic version of the WHOQOL-HIV had a high value of Cronbach's alpha (α > 0.7). However, social relationship had a lower internal consistency (α = 0.57) as compared to others (Table 3).

**Table 3:** Internal consistency of the Amharic version of the WHOQOL-HIV questionnaire

Domain	Coefficient for internal consistency (Cronbach's alpha)
Physical	0.77
Psychological	0.72
Social	0.57
Environmental	0.85
Level of independence	0.76
Spiritual	0.73

Inter domain correlations showed that there were statistically significant associations between domains. However, a weak correlation was observed between the environmental domain and spiritual health (Table 4).

**Table 4:** Correlation between the domains of the Amharic version of the WHOQOL-HIV questionnaire

Domain	PH	Psy	Soc	Env	Ind	Spir
PH	1					
Psy	0.57 *	1				
Soc	0.51 *	0.57 *	1			
Env	0.48 *	0.33 *	0.48 *	1		
Ind	0.76 *	0.52 *	0.50 *	0.55 *	1	
Spir	0.46 *	0.56 *	0.39 *	0.21 *	0.35 *	1

* P < 0.01, PH = Physical health, Psy = psychological health, Soc = Social relationship, Env = Environment, Ind = level of independence, Spir = Spiritual health

We found strong correlation between the QOL domains and the Kessler Scale. Strong correlation was observed between the Psychological domain and the Kessler scale (correlation coefficient, r = -0.59, P = 0.001). Physical, level of independence, spiritual, social and environmental domains had a correlation coefficient of -0.56, -0.54,-0.45,-0.43 and -0.34 with the Kessler scale respectively (P-value = 0.001). Stigma had also statistically significant negative correlation with the spiritual (r = -0.45, P-value = 0.001), psychological (r = -0.33, P-value = 0.001) and social (r = -0.26, P-value = 0.001) domains of QOL.

### Quality of life

After controlling for potential confounding variables like age, sex, occupation, CD4 lymphocyte count, WHO staging and social support, co-infected patients had a lower mean/median score in all domains indicating poor QOL. Mean scores for physical health, social relationship and environmental health among co-infected patients were 13.26(SD = 4.3), 12.15(SD = 3.1) and 11.7(SD = 3.6) respectively (Table 5).

**Table 5:** Comparison of Quality of life of HIV infected patients with and without TB in 3 hospitals of the Oromiya region, Ethiopia

Quality of life Domain	HIV TB co-infection (n = 124) Mean(SD)	HIV without TB (n = 467) Mean(SD)	P-Value
Physical Health	13.26(4.3)	16.81(2.8)	0.001
Psychological Health	14.99(3.2)	16.20(2.5)	0.001
Social relationship	12.15(3.6)	13.64(2.8)	0.001
Environmental Health	11.58(3.1)	12.41(2.7)	0.001
Level of independence	11.7(3.6)	14.98(2.8)	0.001
Spiritual health	16.46(3.9)	17.88(2.8)	0.001

### Predictors of QOL

Depression, having a source of income and family support were strongly associated with most of the QOL domains. In co-infected patients, individuals with depression were 8.8 times more likely to have poor physical health as compared to individual who had no depression, OR = 8.8(95%CI: 3.2, 23). Similarly those without family support were 1.5 more likely to have poor physical health in co-infected and mono-infected patients (Table 6). Similarly, depression, family support and having a source of income were strongly associated with psychological health (Table 7). Among co-infected patients, depressed individuals were 5 times more likely to have poor social relationships as compared to individual without depression, [OR = 5.3, (95%CI: 2.3, 14.2)]. Depression was also associated with poor quality of the social QOL domain among HIV patients OR = 2.4, (95%CI: 1.6, 3.6)]. Family support was associated with social relationships in HIV patients with and without co-infection (P < 0.001). Educational status was significantly associated with the environmental QOL domain. Literate individuals were 4 times more likely to have good QOL as compared to illiterate ones, OR = 4, (95% CI: 2.3, 7.3). High perceived stigma was associated with poor psychological health in TB/HIV co-infected and HIV patients (P < 0.05).

**Table 6:** Determinants of the physical health of HIV infected patients with and without TB in 3 hospitals of Oromiya region, Ethiopia

	Physical health					
**Variables**	**TB/HIV co-infection (n = 124)**		**Adjusted OR(95%CI)**	**HIV without TB (n = 476)**		**Adjusted OR(95% CI)**
	**Good** **N (%)**	**Poor** **N (%)**		**Good** **N (%)**	**Poor** **N (%)**	
Depression						
Yes	7(36.8)	12(63.2)	8.8(3.2,23)	59(27)	159(73)	6.9(4.6,10.4)
No	22(51.2)	23(48.8)	1	181(72.6)	68(27.4)	1
Source of income						
Yes						
No	21(35) 8(14.8)	49(65) 46(85.2)	1 1.7(0.6,4.7)	188(54.8) 52(41.9)	155(45.2) 72(58.1)	1 1.7(1.1,2.6)
Family support						
Yes	15(28.3)	38(71.7)	1	86(56.2)	67(43.8)	1
No	14(19.7)	57(81.3)	1.6(0.6,4)	159(49.8)	160(50.2)	1.5(1.0,2.3)

**Table 7:** Determinants of the Psychological health of HIV infected patients with and without TB in selected hospitals of Oromiya Region, Ethiopia

	Psychological health					
**Variables**	**TB/HIV coinfection (n = 124)**		**Adjusted OR(95%CI)**	**HIV without TB (n = 467)**		**Adjusted OR(95% CI)**
	**Good** **N (%)**	**Poor** **N (%)**		**Good**	**Poor**	
**Depression**						
Yes	20(25.3)	59(74.7)	7.8(3.3,18.7)	85(38.9)	133(41.4)	6.1(3.9, 9.0)
No	32(71.1)	13(28.9)	1	197(79.1)	52(30.9)	1
**Source of income**						
Yes	31(44.3)	39(55.7)	1	221(64.2)	123(35.8)	1
No	21(38.8)	33(61.2)	1.2(0.5, 2.8)	61(49.5)	62(50.5)	1.8(1.1,2.8)
**Family support**						
Yes	28(52.8)	25(47.2)	1	101(66.0)	52(34.0)	1
No	24(33.8)	47(66.2)	2.7(1.1, 6.4)	181(57.6)	133(42.4)	1.5(1.0,2.3)

## Discussion

We compared the QOL of persons with HIV infection with and without active TB. The Amharic version of the WHOQOL-HIV instrument had a good internal consistency to assess the QOL of our TB/HIV co-infected patients. The instrument had strong inter domain and negative correlation with the Kessler scale and the stigma instrument. Strong correlation between the Kessler scale and the psychological domain of the QOL instrument indicated that the two instruments had measured the same concept. Although detail validity study was not done, the above information could indicate that the Amharic Version of the WHOQOL-HIV had good construct validity. The WHOQOL-HIV instrument was previously reported to have a good reliability and validity in different cultures worldwide [26-28]. In this study, co-infected patients had a lower QOL in all of the domains of the WHOQOL-HIV as compared to people living with HIV without TB. The occurrence of two stigmatizing diseases can decrease the QOL by affecting the physical, social and mental wellbeing of the person. In other studies, it was reported that HIV patients had a lower QOL as compared to the general population [13] and that TB patients had a lower QOL as compared to their neighbors [17,18].

Different studies identified several factors which affect the QOL of patients. In a multi-country study among patients with HIV, it was found that women, older age groups, and the less educated had a lower QOL [8]. A study conducted among African American HIV positive participants showed that stigma and presence of symptoms of HIV were associated with poor QOL [9]. In our study, depression and lower income were associated with the physical, social and environmental domains of QOL. Depression can decrease QOL [29] but can also be the result of a poor QOL. Because of the cross sectional nature of our study, we couldn't establish a cause effect relationships between QOL and depression. Perceived stigma was also associated with the psychological domain of QOL. The effect of perceived stigma on QOL was also reported by Yen et al in Taiwan [30].

Lack of social support, lower level of education and income had been reported to be associated with poor QOL of TB patients [15,18]. In our study, income, depression and lack of family support were predictors of poor QOL among TB/HIV co-infected. Participants without adequate income and family support might have a poor nutritional and immune status which in turn could affect the QOL.

In contrast with other studies, we couldn't find an association between CD4 count, WHO staging and other socio-demographic characteristics with QOL [8,15,18].

The results of our study have to be interpreted with caution. Indeed, although the Amharic version of the WHOQOL instrument was used previously, the content and criterion validity of the instrument was not assessed.

## Conclusion

TB/HIV co-infected patients had a poor QOL in all domains of the WHOQOL-HIV instrument. Depression, income and family support were strongly associated with QOL. TB control programs should design strategies to improve the QOL of TB/HIV patients. Depression and self-stigma should be targeted for interventions to improve the QOL. To maximize family support and QOL, families of the patients should be counseled and educated.

## Competing interests

The authors declare that they have no competing interests.

## Authors' contributions

AD conceived the study and was involved in the design, analysis and report writing. MT participated in the design and reviewed the article. YH was involved in report writing and reviewing. NN was involved in report writing. SD and AW were involved in field work and reviewed the article. TB was involved in proposal development and report writing. LA and RC participated in the design and critically reviewed the article. All authors read and approved the final manuscript.
